# A Low Life’s Simple 7 Score Is an Independent Risk Factor for Postoperative Delirium After Total Knee Arthroplasty

**DOI:** 10.3390/medicina61040733

**Published:** 2025-04-15

**Authors:** Yong-Bum Joo, Young-Mo Kim, Woo-Yong Lee, Young-Cheol Park, Jae-Young Park, Chang-Sin Lee

**Affiliations:** 1 Department of Orthopedic Surgery, Chungnam National University Hospital, Chungnam National University College of Medicine, Munhwa-dong, Jung-gu, Daejeon 35015, Republic of Korea; longman76@hanmail.net (Y.-B.J.); osdr69@gmail.com (Y.-M.K.); studymachine@daum.net (W.-Y.L.); ycyeh0830@hanmail.net (Y.-C.P.); 01048406012a@cnuh.co.kr (C.-S.L.); 2 Department of Orthopedic Surgery, Daejeon Eulji Medical Center, Eulji University School of Medicine, 95, Dunsanseo-ro, Seo-gu, Daejeon 35233, Republic of Korea

**Keywords:** osteoarthritis, total knee arthroplasty, postoperative delirium, risk factor, Life’s Simple 7 score

## Abstract

*Background and Objectives*: Postoperative delirium (PODil) is a cognitive condition characterized by sudden fluctuations in consciousness and orientation after surgery. PODil following total knee arthroplasty (TKA) is associated with prolonged hospital stays and increased morbidity. Therefore, prevention of PODil is particularly important. Life’s Simple 7 score, published by the American Heart Association, is a new measure of cardiovascular health (CVH). Better CVH is associated with a lower risk of cognitive impairment. Hence, this study aimed to determine whether Life’s Simple 7 score is associated with PODil following TKA. *Materials and Methods*: This retrospective study included 973 patients who underwent TKA between January 2015 and January 2020. Patients were divided into two groups (group I: delirium group, *n* = 60; group II: non-delirium group, *n* = 913). Demographic data, use of analgesics, surgical factors, underlying diseases, laboratory results, and Life’s Simple 7 score were evaluated. *Results*: Significant differences were observed between the two groups for Parkinson’s disease, intraoperative hypotension, preoperative duloxetine administration, and Life’s Simple 7 score. In the receiver operating characteristic (ROC) curve analysis, the optimal cut-off value for Life’s Simple 7 score was determined to be 8 at the maximal Youden index, with an area under the curve (AUC) of 0.82, a sensitivity of 0.92, and a specificity of 0.58. *Conclusions*: Lower Life’s Simple 7 score is an independent risk factor for the incidence of PODil after TKA. Given its ease of measurement, Life’s Simple 7 score may be a useful measure for predicting PODil and will aid in preoperative risk assessment and post-operative patient management.

## 1. Introduction

Total knee arthroplasty (TKA) is one of the most effective and successful surgeries for treating degenerative osteoarthritis of the knee [1,2,3]. However, several complications can occur after TKA, including postoperative delirium (PODil). The reported prevalence of PODil after TKA ranges from 0.54% to 18.4% [4,5,6,7]. PODil is a cognitive disorder characterized by sudden fluctuations in consciousness and orientation (i.e., inaccurate perception of date, place, and person) after surgery [8]. It is a serious condition that can have adverse clinical and economic outcomes, including an extended hospital stay, increased medical expenses, delayed recovery of normal function, and increased morbidity and mortality [9,10]. Therefore, identifying pre-existing risk factors for PODil is a crucial aspect of perioperative care.

The etiology of PODil is multifactorial, with various precipitating and predisposing factors such as advanced age, male sex, polypharmacy, low levels of hemoglobin or hematocrit, electrolyte imbalances, infection, cardiovascular disease (CVD), and preoperative cognitive impairment [11,12,13,14,15]. However, predicting the occurrence of PODil remains challenging [16], and despite its clinical significance, no reliable tool is currently available for its prediction. Therefore, there is a need for a reliable method to assess the risk of PODil.

Life’s Simple 7 score, a new index for evaluating cardiovascular health (CVH), was published by the American Heart Association in 2010 to determine the metrics needed to monitor and improve CVH status [17]. It comprises seven components: smoking, healthy diet score, body mass index (BMI), physical activity, blood pressure, fasting glucose, and total cholesterol (Table 1) [17]. For each component, a score of 0–2 is assigned according to each criterion, and the scores are summed. A score of 0–6 is considered as low, 7–8 is considered as the middle range, and 9–14 is considered as high, thereby suggesting the standard for CVH [18]. A higher Life’s Simple 7 score corresponds to better CVH, which is associated with a lower incidence of CVD [19,20,21] and CVD is an important factor that increases complications and mortality after TKA. [22] CVH also plays an important role in brain health [23], and better CVH is associated with a lower risk of cognitive impairment [18]. Therefore, a low Life’s Simple 7 score relates to a high risk of CVD and cognitive impairment. However, to the best of our knowledge, no previous studies have evaluated the association between Life’s Simple 7 score and PODil.

Therefore, in this retrospective study, we aimed to determine whether Life’s Simple 7 score is associated with PODil after TKA and identify the additional risk factors for PODil after TKA. We hypothesized that patients with a lower Life’s Simple 7 score would have a higher incidence of PODil.

## 2. Materials and Methods

This retrospective study was approved by the Institutional Review Board (IRB) of our institution (CNUH 2021-09-060). We enrolled 1061 patients who underwent TKA performed by two senior surgeons at our institute between January 2015 and January 2020. Patients who met the following criteria were excluded: To control for variables such as operative time, intraoperative blood loss, and postoperative pain, patients who underwent simultaneous bilateral TKA (*n* = 28), revision TKA (*n* = 53), had multiple trauma (*n* = 5), or had metastatic malignant tumors (*n* = 2). Finally, 973 of the 1061 eligible patients were included in this study.

**Figure 1:**
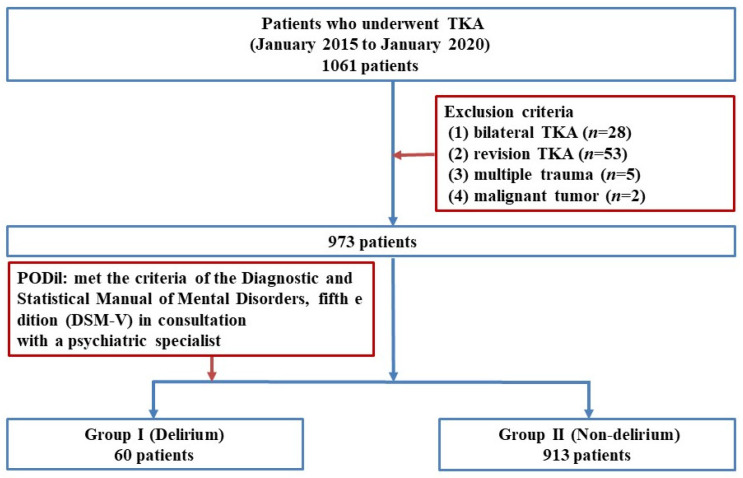
Flow diagram illustrating patient selection. TKA: Total knee arthroplasty; PODil: Postoperative delirium; DSM: Diagnostic and Statistical Manual of Mental Disorders.

To diagnose PODil, we consulted psychiatric specialists at our institution when patients exhibited symptoms such as disturbances in attention and awareness or cognitive impairment (e.g., memory deficits, disorientation, language difficulties, or perceptual disturbances) during hospitalization [24]. The psychiatric specialists assessed the patients and diagnosed delirium if their symptoms met the diagnostic criteria for delirium outlined in the Diagnostic and Statistical Manual of Mental Disorders, Fifth Edition. Based on these diagnoses, patients were categorized into two groups: the delirium group (Group I, *n* = 60) and the non-delirium group (Group II, *n* = 913) (Figure 1). In this study, psychiatric consultations were conducted between the day of surgery and 10 days postoperatively, with an average consultation occurring at 2.83 days postoperatively.

Patients diagnosed with PODil were managed, when necessary, with antipsychotic medications prescribed by a psychiatrist, as well as correction of electrolyte imbalances and anemia. Environmental modifications included placement in a private room with reduced noise levels and allowing close family members to stay for supportive care.

The following data were evaluated through a review of electronic medical records: demographic data (age at TKA, sex, height, and body weight); alcohol history (number of drinks per week); American Society of Anesthesiologists grade [25]; Charlson comorbidity index (CCI) [26]; underlying diseases, including hypertension (HTN), arrhythmia, chronic obstructive pulmonary disease, asthma, diabetes mellitus (DM), history of cerebral infarction, myocardial infarction, renal disease, liver disease, solid tumors, dementia, Parkinson’s disease, and insomnia; use of analgesics (preoperative duloxetine administration, preoperative gabapentin or pregabalin administration, preoperative and postoperative opioid administration, and postoperative patient-controlled analgesia [fentanyl + ramosetron]); surgical factors (type of anesthesia [general anesthesia, spinal anesthesia], operation time [measured from the start of incision to the end of skin closure, minutes], intraoperative hypotension [defined as systolic blood pressure < 90 mmHg for at least 10 min [27]], intraoperative hypothermia [defined as body temperature < 36 °C during surgery [28]], and postoperative transfusion.

Preoperative laboratory data, including the levels of hemoglobin, hematocrit, albumin, sodium (Na), potassium (K), chloride (Cl), calcium (Ca), phosphate (P), blood urea nitrogen (BUN), and creatinine (Cr), were evaluated. Life’s Simple 7 score was assessed by a researcher who was not involved in the surgery.

### Statistical Analysis

Statistical analyses were conducted using SPSS version 26 (SPSS Inc., Chicago, IL, USA). Univariate analysis was performed to identify significant differences between the groups, with statistical significance set at *p* < 0.05. The Chi-square test and Fisher’s exact test were used to analyze correlations between categorical variables and PODil. The t-test was used for continuous variables. Univariate and multivariate logistic regression analyses were performed to identify risk factors for PODil. Odds ratios (OR) and 95% confidence intervals (CI) were calculated to assess relative risks. The area under the curve (AUC) of receiver operating characteristic (ROC) curve was used to evaluate the accuracy of Life’s Simple 7 score in predicting the incidence of PODil, and the Youden index was used to determine the optimal cut-off value.

## 3. Results

The incidence of PODil after TKA was 6.2% (60/973). Table 2 outlines several differences in demographic data and patient characteristics between the two groups. Age at TKA, sex (male), CCI, HTN, DM, renal disease, solid tumors, Parkinson’s disease, and insomnia among underlying diseases were found to be significant factors related with PODil. Additionally preoperative duloxetine administration, type of anesthesia, intraoperative hypotension, and intraoperative hypothermia were identified as significant factors.

Among the preoperative laboratory test results, BUN and Cr levels were significantly higher in group I. The Life’s Simple 7 score was significantly higher in group II (*p* < 0.001).

However, multivariate logistic regression analysis showed significant differences in age at TKA, Parkinson’s disease, preoperative duloxetine administration, intraoperative hypotension, and Life’s Simple 7 scores (Table 3).

Among them, the largest OR was obtained for age at TKA (OR, 1.074), followed by Life’s Simple 7 score (OR, 0.446), intraoperative hypotension (OR, 0.194), preoperative duloxetine administration (OR, 0.171) and Parkinson’s disease (OR, 0.126) (Table 3).

**Figure 2:**
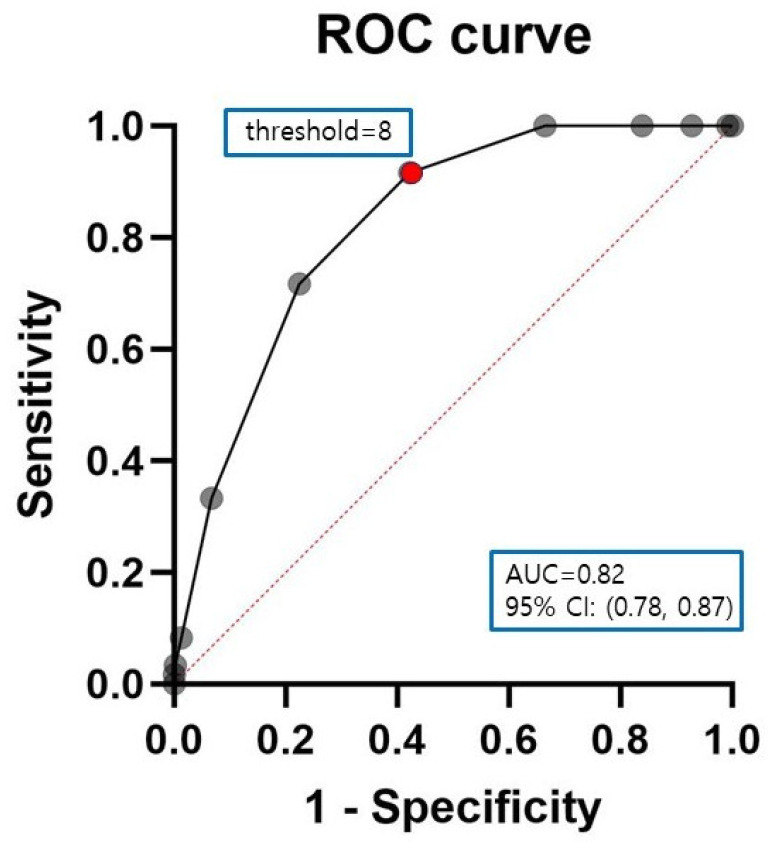
Receiver-operating characteristic (ROC) curves for Life’s Simple 7 score. The area under the curve, cut-off value, Youden’s index, sensitivity, and specificity of Life’s Simple 7 score were 0.82 (0.78–0.87), 8, 0.50, 0.92, and 0.58, respectively (*p* < 0.0001). Black solid line: ROC curve, red dotted line: reference line, black dot: Life’s Simple 7 score, red dot: cut-off value.

In the ROC curve analysis, The Life’s Simple 7 Score cut-off values were determined to be 8 at maximal Youden index, associated with AUC of ROC curve of 0.82 (95% CI 0.78–0.87), a sensitivity of 0.92, and a specificity of 0.58 (Figure 2).

## 4. Discussion

In this study, we determined whether Life’s Simple 7 score is associated with PODil after TKA and identified the additional risk factors for PODil after TKA.

Our findings showed that the incidence of PODil after TKA was 6.2% (60/973), which differs from previous studies by Wang et al. (18.5%, 49/265) [4], Kim et al. (6.0%, 19/318) [5], and Huang et al. (0.6%, 6/1016) [7]. These differences may be due to the subjective nature of the diagnostic criteria for delirium. When a patient exhibits mild symptoms, the diagnosis of delirium may vary depending on the psychiatrist evaluating the patient. Moreover, the differences in results may be attributed to differences in the population pool between studies.

The risk factors for PODil after TKA include advanced age, high body weight, male sex, preoperative dementia and cerebrovascular disease, prolonged hospitalization, delayed ambulation, chronic opioid use, low preoperative hemoglobin level, low preoperative protein level, high postoperative BUN level, and intraoperative hypotension [4,5,6,7].

In the present study, age at TKA, Life’s Simple 7 score, intraoperative hypotension, preoperative duloxetine administration, and Parkinson’s disease were identified as independent risk factors for PODil following TKA.

The factor with the highest OR obtained in this study was the patients’ age at the time of TKA. In older patients, age-related declines in brain function lead to anatomical changes such as decreased synapses in neurons and altered neurotransmitters levels, resulting in memory and concentration difficulties that can contribute to delirium symptoms [4].

In this study, intraoperative hypotension and underlying Parkinson’s disease were also risk factors for PODil after TKA. However, previous studies have reported mixed results regarding these associations. According to Papadopoulos et al. [29], hypotension is associated with decreased cerebral blood flow, and inadequate cerebral oxygenation may cause delirium. However, Hirsch et al. [30] reported that hypotension is not significantly associated with PODil. Preoperative duloxetine administration was associated with increased PODil. Although no studies have shown that duloxetine has a direct effect on the development of delirium, according to Goldstein [31], patients receiving a serotonin reuptake inhibitor have reported mental status changes such as delirium. Parkinson’s disease has also been suggested as a risk factor for PODil in some studies [32,33], but not in others [34]. However, Parkinson’s disease is associated with increased risks of medical and surgical complications [35,36], which suggests that it may also contribute to PODil.

In accordance with our hypothesis, Life’s Simple 7 score was identified as a risk factor for PODil after TKA, with the second highest OR. Life’s Simple 7 score, which can predict CVH, is an independent risk factor for PODil after TKA. However, among the components constituting it, the relationship between the occurrence of PODil and some components is controversial. According to Bjoro et al. [37], a higher BMI, which results in a low Life’s Simple 7 score, is known to be a risk factor for delirium. Zakriya et al. [14] reported that hypertension, DM, and smoking, which lead to low Life’s Simple 7 scores, are unrelated to the occurrence of delirium. Although some of the Life’s Simple 7 score components are unrelated to PODil, Life’s Simple 7 score is an index that evaluates CVH by scoring each component. Based on the results of several previous studies, there is a consensus that CVD increases the incidence of PODil [14,34,38], which is consistent with the results of our study. Additionally, we determined that a Life’s Simple 7 score of 8 was the optimal cut-off value for predicting PODil. The accuracy of this cut-off value was validated using ROC curve analysis, yielding an AUC of 0.82.

Based on our results, Life’s Simple 7 score can be evaluated as an independent risk factor for PODil after TKA. Due to its simplicity of measurement, we expect Life’s Simple 7 score to make an important contribution to the prediction of PODil and greatly aid in preoperative risk assessment and post-operative patient management.

The limitations of this study are as follows: As this was a retrospective study, we were unaware of any missing medical records. Second, since the patient group included in this study was distributed over a period of almost 6 years (2015–2020), three different psychiatrists diagnosed the patients with PODil over the course of the study. Third, a sex bias may exist because most patients who underwent TKA were female (male, 135; female, 831). However, we found that sex was not a factor associated with PODil after TKA. Fourth, the preoperative state of cognitive dysfunction was not baselined before the surgery. While most patients were alert preoperatively, further research is needed to examine the correlation between preoperative cognitive status and the incidence of PODil. Additionally, this study included only a limited set of chronic conditions and medication histories. Future investigations should incorporate a broader preoperative baseline assessment, including cognitive function and chronic disorders, to enhance our understanding of PODil risk factors.

## 5. Conclusions

Lower Life’s Simple 7 score is an independent risk factor for the incidence of PODil after TKA. Due to its simplicity of measurement, Life’s Simple 7 score may be a useful measure for predicting PODil and will aid in preoperative risk assessment and post-operative patient management. Other risk factors for PODil after TKA include age at TKA, Parkinson’s disease, intraoperative hypotension, and preoperative duloxetine administration.

## Figures and Tables

**Table 1:** Components of Life’s Simple 7 Score [18].

Component	2 Points	1 Point	0 Point
Smoking	Non-smoker or former smoker (>1 year)	Former smoker (≤1 year)	Current smoker
Healthy diet score *	4–5 points	2–3 points	0–1 point
Body mass index	<25 kg/m^2^	25–29.9 kg/m^2^	≥30 kg/m^2^
Physical activity	≥4 times per week of intense physical activity, with sweating	1–3 times per week of intense physical activity, with sweating	No intense physical activity; no sweating
Blood pressure	<120/80 mmHg	Systolic blood pressure 120–139 mmHg or diastolic blood pressure 80–89 mmHg or treated to an ideal level	Systolic blood pressure ≥ 140 mmHg or diastolic blood pressure ≥ 90 mmHg
Fasting glucose	<100 mg/dL	100–125 mg/dL or treated to an ideal level	≥240 mg/dL
Total cholesterol	<200 mg/dL	200–239 mg/dL or treated to an ideal level	≥240 mg/dL

Life’s Simple 7 score can range from a minimum of 0 to a maximum of 14 points. Low: 0–6 points, Middle: 7–8 points, High: 9–14 points. * The American Heart Association defines the five components of a healthy diet as follows: “(1) fruits and vegetables: ≥4.5 cups per day; (2) fish: ≥two or more 3.5-oz servings per week (preferably oily fish); (3) fiber-rich whole grains (≥1.1 g of fiber per 10 g of carbohydrate): ≥three or more 1-oz-equivalent servings per day; (4) sodium: <1500 mg per day; and (5) sugar-sweetened beverage: ≤450 kcal (36 oz) per week” [18].

**Table 2:** Comparison of demographic data and characteristics of patients with/without postoperative delirium.

Factors	Group I (*n* = 60) Delirium	Group II (*n* = 913) (Non-Delirium)	*p* Value
Demographic Data			
Age at TKA (years)	74.48 ± 5.20	69.98 ± 7.08	<0.001 *
Sex			0.001 *
Male	15	120	
Female	40	791	
Height (cm)	152.64 ± 9.56	152.70 ± 7.26	0.960
Body weight (kg)	62.29 ± 11.27	62.23 ± 10.25	0.963
Characteristics			
Alcohol (frequency per week)			0.067
<3	53	892	
3–4	2	4	
>4	0	10	
American Society of Anesthesiologists grade			0.370
1	2	14	
2	39	706	
3	14	191	
Charlson Comorbidity Index	5.40 ± 1.73	4.22 ± 1.38	<0.001 *
Underlying diseases			
Hypertension (%)	47 (85.5)	624 (68.5)	0.003 *
Arrhythmia (%)	1 (1.8)	30 (3.3)	0.716
Chronic obstructive pulmonary disease (%)	1 (1.8)	14 (1.5)	1.000
Asthma (%)	1 (1.8)	17 (1.9)	1.000
Diabetes mellitus (%)	23 (41.8)	215 (23.6)	<0.001 *
Cerebral infarction (%)	7 (12.7)	72 (7.9)	0.144
Myocardial infarction (%)	4 (7.3)	53 (5.8)	0.088
Renal disease (%)	6 (10.9)	30 (3.3)d	0.019 *
Liver disease (%)	4 (7.3)	27 (3.0)	0.118
Solid tumor (%)	6 (10.9)	43 (4.7)	0.029 *
Dementia (%)	2 (3.6)	8 (0.9)	0.122
Parkinson’s disease (%)	4 (7.3)	8 (0.9)	0.001 *
Insomnia (%)	1 (1.8)	1 (0.1)	<0.001 *
Analgesics			
Preoperative duloxetine (%)	17 (30.9)	101 (11.1)	<0.001 *
Preoperative gabapentin or pregabalin (%)	10 (18.2)	192 (21.1)	0.907
Preoperative opioid (%)	37 (67.3)	534 (58.6)	0.137
Postoperative opioid (%)	49 (89.1)	772 (84.7)	0.262
Postoperative PCA (%)	54 (98.2)	891 (97.8)	0.610
Surgical factors			
Type of anesthesia			<0.008 *
General	49	878	
Spinal	6	33	
Operation time (minute)	82.97 ± 17.32	82.60 ± 16.11	0.975
Intraoperative hypotension (%)	12 (22)	57 (6.3)	0.001 *
Intraoperative hypothermia (%)	11 (20.0)	59 (6.5)	0.002 *
Postoperative transfusion (%)	15 (27.2)	246 (27.0)	1.000
Preoperative laboratory results			
Hemoglobin (g/dL)	12.1 ± 1.50	12.2 ± 1.38	0.447
Hematocrit (%)	35.94 ± 4.44	36.42 ± 4.00	0.371
Albumin (g/dL)	3.79 ± 0.46	3.88 ± 0.39	0.149
Sodium (Na) (mEq/L)	140.56 ± 2.73	140.70 ± 3.01	0.720
Potassium (K) (mEq/L)	4.15 ± 0.37	4.13 ± 0.40	0.795
Chloride (Cl) (mEq/L)	103.80 ± 2.62	104.28 ± 3.38	0.285
Calcium (Ca) (mg/dL)	8.81 ± 0.57	8.91 ± 0.59	0.191
Phosphate (P) (mg/dL)	3.54 ± 0.59	3.62 ± 0.66	0.386
Blood urea nitrogen (BUN) (mg/dL)	17.67 ± 5.86	15.86 ± 5.41	0.013 *
Creatine (Cr) (mg/dL)	0.82 ± 0.23	0.72 ± 0.47	0.003 *
Life’s Simple 7 score	6.88 ± 1.25	8.85 ± 1.66	<0.001 *

* Represents a significant outcome (*p* < 0.05). mean ± standard deviation. PCA, patient-controlled analgesia; TKA, total knee arthroplasty.

**Table 3:** Multivariable logistic regression analysis of the risk factors for delirium.

Factors	Odds Ratio	Odds Ratio 95% CI		*p* Value
		Low	High	
Age at TKA	1.074	1.005	1.148	0.035 *
Sex	0.699	0.298	1.638	0.410
CCI	1.351	0.948	1.927	0.096
Hypertension	0.606	0.233	1.574	0.304
Diabetes mellitus	1.860	0.647	5.346	0.249
Renal disease	0.902	0.229	3.560	0.883
Solid tumor	0.532	0.131	2.151	0.376
Parkinson’s disease	0.126	0.022	0.713	0.019 *
Insomnia	0.039	0.001	1.872	0.100
Preoperative duloxetine	0.171	0.072	0.405	<0.001 *
Type of anesthesia	2.434	0.544	10.893	0.245
Intraoperative hypotension	0.194	0.075	0.505	0.001 *
Intraoperative hypothermia	0.496	0.178	1.384	0.180
BUN	0.967	0.898	1.042	0.379
Cr	1.643	0.661	4.089	0.285
Life’s Simple 7 score	0.446	0.331	0.601	<0.001 *

* Represents a significant outcome (*p* < 0.05). BUN, blood urea nitrogen; CCI, Charlson Comorbidity Index; CI, confidence interval; Cr, creatine; TKA, total knee arthroplasty.

## Data Availability

The data generated and/or analyzed in this study are included within this article.

## Abbreviations

The following abbreviations are used in this manuscript:

TKA	Total knee arthroplasty
PODil	Postoperative delirium
CVD	Cardiovascular disease
CVH	Cardiovascular health
BMI	Body mass index
IRB	Institutional Review Board
CCI	Charlson comorbidity index
HTN	Hypertension
DM	Diabetes mellitus
BUN	Blood urea nitrogen
OR	Odds ratio
CI	Confidence interval
AUC	Area under the curve
ROC	Receiver operating characteristic
